# The Mitochondrial Unfolded Protein Response as a Non-Oncogene Addiction to Support Adaptation to Stress during Transformation in Cancer and Beyond

**DOI:** 10.3389/fonc.2017.00159

**Published:** 2017-07-26

**Authors:** Timothy C. Kenny, Giovanni Manfredi, Doris Germain

**Affiliations:** ^1^Department of Medicine, Division of Hematology/Oncology, Icahn School of Medicine at Mount Sinai, Tisch Cancer Institute, New York, NY, Unites States; ^2^Feil Family Brain and Mind Research Institute, Weill Cornell Medicine, New York, NY, Unites States

**Keywords:** mitochondria, mitochondrial unfolded protein response, cancer, ALS, sirtuin deacetylase, estrogen receptor

## Abstract

Upon accumulation of misfolded proteins in the mitochondria, the mitochondrial unfolded protein response (UPR^mt^) is activated. This review focuses on the role of this response in cancer. We discuss evidence that during transformation, the UPR^mt^ may play an essential role in the maintenance of the integrity of the mitochondria in the face of increased oxidative stress. However, the role of the UPR^mt^ in other diseases is also emerging and is therefore also briefly discussed.

## Introduction

The proper folding of proteins is fundamental to cellular life. Perturbations to this process promote the formation of protein aggregates and underlie a number of human pathologies.

Aggregation of proteins in the lumen of organelles represents an additional challenge, as they cannot be directly presented to the ubiquitin/proteasome system. The discovery of the endoplasmic reticulum mediated degradation was originally made in yeast and revealed the first mechanism of how misfolded proteins in the lumen of the endoplasmic reticulum can be retro-translocated to the cytoplasm and ubiquitinated for their degradation by the proteasome [for a recent review (1)]. Further, the accumulation of misfolded proteins in the lumen of the endoplasmic reticulum was found to lead to the transcription of the endoplasmic reticulum chaperone, BiP (KAR2) (2). Using a genetic screen, the same authors identified inositol-requiring transmembrane kinase/endonuclease (IRE1) as the first sensor of the UPR^ER^, which communicates the proteotoxic stress to the nucleus (3, 4). While IRE1 is the sole sensor of the UPR^ER^ in yeast, higher eukaryotes have three distinct axes of the UPR^ER^—IRE, PERK, and ATF6, all of which represent independent signaling cascades that activate separate pathways to cumulatively reduce proteotoxic stress and maintain organelle homeostasis (5, 6). The study of the UPR^ER^ identified CHOP as a transcription factor essential for this response. More recently, a similar mechanism has been proposed to take place for the elimination of misfolded proteins inside the mitochondria (7, 8). The initial axis of the mitochondrial unfolded protein response (UPR^mt^) to be discovered also implicates CHOP (8). However, as described in details below, the effect of CHOP is context dependent and there is no overlap between the UPR^ER^ and UPR^mt^ in that stress in the lumen of the endoplasmic reticulum do not activate the UPR^mt^ and vice versa.

Further, in contrast to the endoplasmic reticulum, the mitochondrial matrix is rich in chaperones and proteases and is therefore well equipped to manage the accumulation of misfolded proteins. In fact, an even more recent study described a critical role of the mitochondria in the management of cytoplasmic proteostasis (9). In this study, the authors found that, upon heat shock stress in yeast, cytosolic proteins that are prone to aggregation are imported into the mitochondria for degradation (9), a phenomenon they named MAGIC (mitochondria as guardian in cytosol). While this concept is intriguing, how misfolded proteins are transported from the cytoplasm into the mitochondria remains unclear. One possibility is that cytosolic chaperones may partially unfold misfolded proteins allowing them to enter the mitochondria. Importantly for this review, the same study reported that import of cytosolic misfolded proteins was found in both the inter-membrane space (IMS) and the matrix of the mitochondria. How the localization of misfolded proteins within the mitochondria after import from the cytosol is determined also remains to be determined. Nevertheless, if cytosolic proteins can indeed be imported in both the matrix and the IMS, this would have major implications for the UPR^mt^ since, unlike to matrix, the IMS has limited protein quality control and no heat shock proteins. Therefore, raising the question as to how accumulation of misfolded proteins in the IMS would help alleviate proteotoxic stress.

## Misfolded Proteins in the Mitochondrial IMS

If MAGIC is a conserved mechanism in mammals, it is predicted to lead to the accumulation of misfolded proteins in the IMS, as a result of their import from the cytosol under stress conditions. In addition to import of cytosolic proteins in the mitochondria, accumulation of misfolded proteins in the IMS can also arise within the mitochondria itself. This effect may be especially true in cancer cells that are characterized by increased levels of reactive oxygen species (ROS), which cause oxidation of proteins and their misfolding.

The elevation in ROS in cancer cells combined with the Warburg effect, which refers to the elevation in glycolysis for the production of ATP, have led to the misconception that cancer cells have defective mitochondria. In contrast, it is now recognized that most cancer cells continue to require oxidative phosphorylation. This observation has led to the idea of the reverse-Warburg effect, the recognition of oxidative tumors, and metabolic flexibility [for a recent review (10)]. ROS contribute to the reprogramming of the mitochondrial of cancer cells and have been shown to play a causative role in tumorigenesis and cancer progression (11–13). While elevation in ROS levels benefit cancer cells by promoting genomic instability and metabolic reprogramming, if left uncontrolled leading to excessive levels, ROS can cause severe DNA damage, oxidation of lipids and proteins, and cause cell death (13). Therefore, cancer cells must acquire mechanisms to keep their ROS levels within a window that is compatible with the maintenance of the integrity of the organelle. Given that the primary site of ROS production in the mitochondria is the electron transport chain (ETC) of the mitochondrial inner-membrane, the mitochondria of cancer cells are particularly vulnerable to oxidative stress. Mitochondria are double membrane bound organelles composed of the outer and inner-membranes, thereby creating two sub-compartments. The mitochondrial matrix contains the mitochondrial genome and mitochondrial-specific ribosomes for the translation of mitochondrial encoded proteins as well as a multitude of well-characterized enzymes involved in intermediary metabolism. The IMS, however, has been largely overlooked and is often perceived merely as a storage space for pro-apoptotic proteins, until they are released into the cytosol for the execution of apoptosis. In contrast, over 100 proteins reside in the IMS, representing roughly 10% of the mitochondrial proteome. IMS proteins are actively involved in metabolism, protein import, oxidative protein folding, ETC complex assembly, export of ferrous precursors, and transport of metabolites, metal ions, and lipids (14). Because ETC-generated ROS is produced on both sides of the inner-membrane (15), ROS-mediated misfolding of proteins also occurs in the IMS. In addition, oxidative protein folding, which occurs only in the IMS and the endoplasmic reticulum, is a process by which proteins are folded into proper conformations through the formation of disulfide bonds, and in so doing produce one molecule of ROS per cycle of folding (14). ROS produced by this process can also contribute to the misfolding and aggregation of IMS proteins (15). Therefore, given the fact that the IMS of the mitochondria has very little capacity to handle misfolded protein when compared with that of the mitochondrial matrix, the IMS appears poised for the accumulation of misfolded proteins.

Our group has previously sought to understand the mechanisms by which misfolded proteins in the IMS of the mitochondria are managed and resolved in the context of cancer cells. Using a mutant form of endonuclease G (EndoG), which misfolds and forms protein aggregates in the IMS, we first reported that the proteasome in the cytosol and the protease OMI in the IMS cooperate to limit the accumulation of misfolded proteins in the IMS (16). We proposed that the proteasome acts as a pre-import checkpoint, while OMI acts a post-import checkpoint (16).

## The Brief Summary of the Discovery of the UPR^mt^

The UPR^mt^ was originally identified in mammalian cells using the overexpression of mitochondrial matrix localized misfolded OTCΔ (17). This first axis of the UPR^mt^ was found to be mediated through the transcription factor CHOP leading to the upregulation of a number of mitochondrial chaperones and proteases, such as ClpP, hsp10, and hsp60 (17). This effect was shown to be mediated through binding of CHOP to mitochondrial upstream elements (18). Further, it was shown that binding of CHOP to the promoters of target genes in response to mitochondrial proteotoxic stress was dependent on AP-1 (18–20). Mitochondrial matrix proteotoxic stress did not lead to activation of UPR^ER^ genes despite CHOP being implicated in the UPR^ER^, suggesting that AP-1 provides the context specificity of CHOP.

Since its discovery, much of the work in the field of the UPR^mt^ has been focused on this axis, with particular emphasis on the chaperone hsp60, which has been used extensively as a reporter of the UPR^mt^ in genetic screens to identify players of the UPR^mt^ in Caenorhabditis elegans. Using this model system, ATFS-1 and the DVE-1/UBL5 complex have been identified as important transcriptional activators of the UPR^mt^ (21–33).

**Figure 1:**
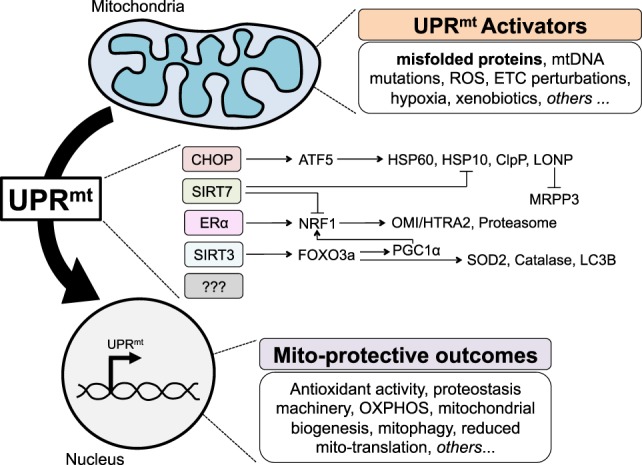
The mammalian mitochondrial unfolded protein response (UPR^mt^), to date. Various perturbations to mitochondria homeostasis, including misfolded proteins, activate currently known axes of the UPR^mt^ to signal to the nucleus in a retrograde fashion, and induce gene expression that results in a number of mito-protective outcomes.

More recently, the transcription factor ATF5 was identified as the mammalian ortholog of ATFS-1 (34). As ATF5 has been shown to act downstream of CHOP (35) and both CHOP and ATF5/ATFS-1 activation leads to induction of mitochondrial chaperones and proteases, CHOP and ATF5 reside in the same axis of the UPR^mt^, which is therefore referred to as the CHOP axis (Figure 1). While a body of literature already exists around the role of ATF5 in cancer biology, notably in the regulation of apoptosis (36), it will be interesting to further investigate the role of ATF5 in the context of the UPR^mt^ and cancer.

The CHOP axis of the UPR^mt^ and its role in cancer biology has been recently addressed, mostly focusing on ClpP, the AAA+ peptidase subunit of the ClpXP, a complex that is induced by CHOP. ClpP was identified using a genetic screen for the viability of leukemic cells (37). The authors then showed that ClpP inhibition had potent antitumor effects both *in vitro* and *in vivo* (37). In a separate study, a proteomic screen for proteins associated with the oncoprotein survivin led to the identification of ClpP (38). Further, ClpP was found to be overexpressed in almost every solid tumor type and analysis of patient databases showed that elevated ClpP expression significantly correlated with worse clinical outcomes (38).

Because of the limited extent of the protein quality control mechanisms in the IMS, we wanted to determine whether the CHOP axis of the UPR^mt^ is also activated upon proteotoxic stress in the IMS. A mutant form of the IMS resident protein EndoG was used as a tool to target misfolded proteins specifically to the IMS. To our surprise, we found that in estrogen receptor alpha (ERα) positive breast cancer cells, accumulation of misfolded proteins in the IMS did not activate the CHOP axis. While confirming in this model system that expression of OTCΔ in the matrix activates CHOP, we found that IMS stress activates AKT, which then phosphorylates the ERα to promote its transcriptional activity in a ligand-independent fashion (39). Further, we found that activation of the ERα following stress in the IMS leads to increased expression of OMI and the activation of the proteasome, therefore linking stress in the IMS to the elements of the protein quality control of the IMS we had previously identified. Moreover, as the ERα was previously shown to activate the transcription of NRF1, a major transcription factor involved in mitochondrial biogenesis (40), we also analyzed NRF1 in our EndoG model. We found that upon IMS stress, the transcription of NRF1 is elevated and dependent on the ERα, as inhibition of the ERα by shRNA prevents activation of NRF1 under these conditions (39).

Given that a significant percentage of breast cancers do not express the ERα, the finding of the ERα axis of the UPR^mt^ raised the question as to how ERα negative breast cancer cells cope with misfolded protein in the IMS. To address this question, we used EndoG to induce stress in the IMS and OTCΔ to induce stress in the matrix in ERα negative breast cancer cells. These studies led to the identification of a third axis of the UPR^mt^ regulated by the mitochondrial NAD-dependent sirtuin deacetylase (SIRT3) (41) (Figure 1). We found that in ERα negative cells, upon stress in either the IMS or the matrix, expression of SIRT3 is elevated and leads to the deacetylation of the transcription factor FOXO3a. While the deacetylation of FOXO3a in response to mitochondrial stress was found to be SIRT3-dependent, it likely occurs through an indirect mechanism, as FOXO3a and SIRT3 have different subcellular localization (41). We reported that deacetylated FOXO3a leads to the translocation of FOXO3a in the nucleus, where it promotes the transcription of target genes SOD2 and catalase (41). In these cells, either IMS-stress or matrix-stress activated CHOP and its downstream targets hsp60 and hsp10. Importantly, within the same year, a very similar axis of the UPR^mt^ was also reported in *C. elegans* and found to influence lifespan (42).

In addition to the antioxidant machinery, we reported that the SIRT3 axis of the UPR^mt^ induces the elimination of irreversibly damaged mitochondria through the process of mitophagy (41). As for SOD2 and catalase, the induction of mitophagy upon accumulation of misfolded proteins in the mitochondria was abolished when SIRT3 was inhibited by shRNA. However, inhibition of SIRT3 did not affect the induction of Hsp60 under these conditions and inhibition of CHOP did not inhibit the expression of SOD2, catalase, or markers of autophagy. We therefore concluded that CHOP and SIRT3 regulate different axes of the UPR^mt^.

While the identification of the CHOP axis by the Hoogenraad group and the ERα and SIRT3 axes by our group was obtained causing accumulation of misfolded proteins directly in the matrix or the IMS, the UPR^mt^ has been found to be activated by other, more indirect, stressors. Notably, inhibition of the ETC, inhibition of mitochondrial translation, or inhibition of matrix chaperones can also activate the UPR^mt^. The use of these alternative stimuli has led to the identification of additional axes. First, in hematopoietic stem cells, SIRT7 has been shown to negatively regulate NRF1 activity and induce CHOP target genes—Hsp60, Hsp10, and ClpP (Figure 1). Second, global transcriptomics and proteomics performed on HeLa cells treated with a number of agents perturbing mitochondrial proteostasis identified a reduction in pre-RNA processing and an inhibition of mtDNA-encoded translation induced by the degradation of MRPP3 (43). The transcription factor responsible for this effect remains unknown and therefore whether MRPP3 is downstream of an already known axis or represents a novel axis remains to be determined (Figure 1).

## Validation of the ERα and SIRT3 Axes of the UPR^mt^ in Other Models

Following the identification of ERα and SIRT3 axes of the UPR^mt^ using EndoG and OTCΔ overexpression as tools to induce stress in the IMS and matrix, respectively, we next aimed at validating these axes under more physiological conditions.

### Validation of the ERα Axis in Familial ALS

The field of neurodegeneration has long appreciated the importance of misfolded proteins, as it has been identified as a common mechanism in a number of human neurodegenerative disorders, such as Alzheimer’s disease, Huntington’s disease, Parkinson’s disease, and ALS (44, 45). While the majority of ALS cases are sporadic, 10% are familial and have been linked to pathogenic mutations in specific genes (46). Superoxide dismutase 1 (SOD1) was the first gene reported to be mutated in familial ALS and over 100 different mutations have been documented (46). Localized in both the cytosol and the IMS of the mitochondria, mutations in SOD1 cause misfolding and subsequent protein aggregation in both cellular compartments (46, 47). Mutant SOD1 aggregates are cytotoxic to the motor neurons of patients with familial ALS and drive disease progression. The SOD1G93A mutation is the best characterized mutation and it has been used to generate the first mouse model of familial ALS. These mice develop muscle atrophy and other symptoms of ALS and die within 130 days. In contrast, in mice where SOD1G93A is targeted exclusively within the IMS and is absent from the cytoplasm, symptoms are drastically reduced and survival prolonged to 360 days (48). This observation led us to postulate that the accumulation of SOD1G93A in the IMS may activate the UPR^mt^. Further, in this model, the absence of the SOD1G93A cytosolic aggregates, which we postulate may mitigate the ability of the UPR^mt^ to protect the integrity of the mitochondria, may explain the longer survival in the IMS targeted model of SOD1G93A.

To test this hypothesis, we recently validated the activation of the ERα axis of the UPR^mt^ in both the untargeted and IMS-targeted mouse models of SOD1G93A familial ALS (49). Interestingly, we found a significant gender difference in the activation of the proteasome as well as OMI (49). Further, in the absence of the ERα, mutant G93A-SOD1 failed to activate this response (39, 49). This finding therefore, does not only validate the ERα axis of the UPR^mt^ in a disease relevant model *in vivo* but it also raises the distinct possibility that sex differences observed in several neurodegenerative diseases may be related to the ERα status of the affected tissue. We will actively pursue this possibility in the future.

### Validation of the SIRT3 Axis in Cancer Cells under Endogenous Level of Mitochondrial Stress

While the use of EndoG and OTCΔ was instrumental in the discovery of several players of the UPR^mt^, in reality the accumulation of misfolded proteins is likely to be present in both the matrix and the IMS as ROS is produced on both sides of the inner-membrane simultaneously. Further, since our hypothesis is that activation of the UPR^mt^ will increase mitochondrial fitness and adaptation to stress, we reasoned that the activation of UPR^mt^ could be linked to a more aggressive cancer phenotype, such as increased invasion capacity. We therefore investigated whether the SIRT3 and CHOP axes of the UPR^mt^ may be linked to the metastatic potential of breast cancer cells.

We found that markers of activation of the SIRT3 axis of the UPR^mt^ could distinguish metastatic from non-metastatic cells in a panel of breast cancer cell lines (50). When overexpressed in non-invasive cells, SOD2, the antioxidant induced by the SIRT3 axis of the UPR^mt^, increased invasion. When SOD2 was inhibited by shRNA, the invasion of normally invasive cells was significantly reduced. Additionally, through the analysis of a collection of cybrids—cells lines with a common nuclear genome, but different mitochondrial genomes—we demonstrated that mitochondrial disease patient-derived mtDNA mutations influence the levels of mitochondrial stress and subsequently the levels of activation of the SIRT3 axis of the UPR^mt^. Activation of the SIRT3 axis of the UPR^mt^ correlated with invasion capacity of the cybrids, further emphasizing the link between this pathway and metastasis. Importantly, activation of the SIRT3 axis of the UPR^mt^ was seen in primary breast cancer patients and high expression, using SOD2 as a marker, was significantly associated with worse disease-free survival (50). Additionally, in a collection of 50 matched primary and metastatic lesions from breast cancer patients, using SOD2 as a marker, we observed a significant increase in activation of the SIRT3 axis of the UPR^mt^, in metastatic lesions when compared with primary lesions. These patient data strongly support our hypothesis that activation of the SIRT3 axis of the UPR^mt^ increases the invasiveness and metastatic potential of cancer cells. In contrast the CHOP axis, monitored using hsp60 as a marker, did not distinguish the metastatic form the non-metastatic cells. Rather, hsp60 was found to be elevated very early after oncogene induction (50). This finding suggests the possibility that the number of axes of the UPR^mt^ engaged in mounting a protective response to mitochondrial stress may increase over disease progression.

## Concluding Remarks and Future Perspectives

In light of the recent findings discussed in this review, it becomes increasingly clear that the UPR^mt^ is a complex transcriptional pathway that expands well beyond the activation of proteases and chaperones of the matrix. We propose that the IMS plays a central role in initiating this pathway. One critical question that remains to be answered is what are the sensors/transducers of the UPR^mt^. Proteins able to translocate from the mitochondria to the nucleus, such as ATF5 (34), are prime candidates but others, such as GPS2 (51–55), which interestingly regulates the activity of the ERα (56), may be implicated. In addition, we cannot exclude the possibility that proteins at the surface of the mitochondria can also act as sensors and transducers of the UPR^mt^ or that signaling cascades such as the one recently described, initiated by Lyn kinase in the IMS by ROS (57), may also be implicated in the UPR^mt^. Additional *in vivo* validation of the UPR^mt^ in mammalian systems in the context of cancer and other pathologies is an important future direction for the new pathway. Further effort should be made to mechanistically integrate the currently known axes of the UPR^mt^ as there is undoubtedly complex orchestration of multiple responses activated in response to mitochondrial stress. Notably, the name mitochondrial stress response has been recently proposed (58). However, misfolded proteins can arise directly or indirectly from a number of mitochondrial perturbagens, including mutation in mitochondrial genome. For instance, bacterial infection by *Psedumonas aeruginosa*, which causes mitochondrial dysfunction and leads to UPR^mt^ activation in *C. elegans* and the transcriptional upregulation of innate immunity genes (31). Therefore, the field may benefit from expanding the term UPR^mt^ to integrated mitochondrial stress response to more fully capture the numerous retrograde signaling cascades that are activated in response to changes in mitochondrial homeostasis, including proteostasis.

## Author Contributions

TK has contributed to the writing of this review and made the figure. GM has contributed to the writing of this review. DG is the corresponding author and has contributed to the writing and finalized this review.

## Conflict of Interest Statement

The authors declare that the research was conducted in the absence of any commercial or financial relationships that could be construed as a potential conflict of interest.
